# Factors associated with unsafe work behaviours in an Iranian petrochemical company: perspectives of workers, supervisors, and safety managers

**DOI:** 10.1186/s12889-020-09286-0

**Published:** 2020-07-31

**Authors:** Azita Zahiri Harsini, Fazlollah Ghofranipour, Hormoz Sanaeinasab, Farkhondeh Amin Shokravi, Philip Bohle, Lynda R. Matthews

**Affiliations:** 1 Department of Health Education, Faculty of Medical Sciences, Tarbiat Modares University, P.O. Box 14115-111, Tehran, Iran; 2 Faculty of Medicine and Health, The University of Sydney, Sydney, Australia; 3 Health Research Center, Lifestyle institute, Baqiyatallah University of Medical Sciences, Tehran, Iran; 4 Tasmanian School of Business and Economics, University of Tasmania, Private Bag 84, Hobart, Tasmania 7001 Australia; 5 Work and Health Research Team, Faculty of Medicine and Health, The University of Sydney, Sydney, Australia

**Keywords:** Safe work behaviours, Occupational health, Petrochemical industry, Workplace accidents, Industrial hazards

## Abstract

**Background:**

The petrochemical industry is hazardous, in part because of the inherently dangerous nature of the work conducted, and incidents frequently result in significant financial and social losses. The most common immediate cause of incidents and injuries in this industry is unsafe worker behaviour. Identifying the factors encouraging unsafe work behaviours is the first step in taking action to discourage them. The aim of this study was to (a) explore workers’, supervisors’ and safety managers’ attitudes and perceptions of safety in a petrochemical company in Iran, and (b) identify the factors that discourage safe work behaviours.

**Methods:**

A qualitative study was conducted by applying the steps described by Graneheim and Lundman (2004). Twenty participants were recruited from an Iranian petrochemical company using a multi-stage approach, with initial purposive sampling followed by snowball sampling to enhance recruitment. Individual face-to-face and semi-structured interviews were conducted to gain an in-depth understanding of factors acting as barriers to safe behaviour. The interviews were recorded and transcribed in Persian and then translated into English. Conventional content analysis was performed.

**Results:**

The main themes emerging from the interviews were: (i) poor direct safety management and supervision; (ii) unsafe workplace conditions; (iii) workers’ perceptions, skills and training; and (iv) broader organisational factors.

**Conclusions:**

The findings give insights into practical organisational measures that can be implemented by management to promote workers’ commitment to safety and engage in safe behaviours in their workplace.

**Trial registration:**

Iranian Registry of Clinical Trials: IRCT20170515033981N2. Retrospectively registered 19 June 2018.

## Background

Unsafe behaviour and human error are important contributors to dangerous incidents and occupational injuries. For example, Shin and colleagues [1] estimate that approximately 88% of workplace incidents in the construction industry are caused by unsafe behaviours, 10% by unsafe physical conditions and 2% by unforeseeable factors or ‘Acts of God’. Encouraging safe behaviour is therefore an important element of improving safety performance [2]. Most behaviour-based safety researchers concentrate predominately on workers’ behaviours that can directly prevent workplace injuries and improve workers’ safety [3]. However, it is not sufficient to focus exclusively on individual behaviour because organizational factors contribute to unsafe behaviours and errors, and directly to injury in some cases [4]. Long working hours and high job demands are good examples of organisational factors; various studies have found that long working hours are associated with higher workplace injury rates and poorer worker productivity and well-being [5]. Increased job demands are associated with more frequent unsafe behaviours [6] and may contribute to injuries by exhausting employees’ mental and physical resources [6, 7]. Unfortunately, a strong focus on analysing the short-term causes of incidents (in the time period immediately before they occur) may obscure the contribution of organisational factors to injuries and dangerous incidents [8].

The growing incidence of occupational injuries and work-related deaths in Iran has raised concern about workers’ health and safety in many organisations. According to the Council of Labor Affairs’ 2010 annual report, the number of workers suffering injuries and sickness was highest in the petrochemical industry, where serious deficiencies in workplace safety, including equipment failures, were identified [9]. The Iranian petrochemical industry is important for both economic and employment reasons. Iran is now the second largest producer and exporter of petrochemicals in the Middle East, with more than 54 petrochemical complexes [10]. Iran has a 2.4% share of global production of petrochemical products in various types of polymeric materials, chemicals, and fertilizers. In addition, the annual rankings of the global top 100 petrochemical companies by the Institute of Cheminformatics Studies show that the Iran National Petrochemical Company ratings improved from 82 in 2004 to 39 in 2011 [11].

The petrochemical industry is the most important and highest-earning in Iran. Petrochemical installations have high levels of risk due to the flammable materials processed and the severe consequences when major incidents occur [8]. They are, because of the working, environmental or geological conditions, prone to dangerous incidents and deaths [12], in which ineffective management practices and, in turn, unsafe work behaviour play important roles. In 2015, Norozi and colleagues [13] reported that, over the preceding 10 years, more than 198 work-related fatalities occurred in Iranian petrochemical companies, principally because ineffective management systems failed to prevent major incidents. These deaths indicate that current management systems must be improved to more effectively prevent major injuries and accidents [13]. To mitigate potential work hazards, it is critical to determine potential risk sources, assess their probabilities and intensities, and manage them effectively [14]. Implementing appropriate measures is not just necessary to reduce the number of dangerous incidents and prevent workplace injuries, but also to improve work productivity and quality [15, 16].

Another critical issue in managing safe behaviour in this industry is the development of educational interventions that minimize workers’ exposure to hazards and related risks. Safety training is an effective strategy for changing unsafe behaviours, discouraging false beliefs about safety, and preventing occupational accidents [17]. However, understanding the key factors affecting safety is a prerequisite to identifying training needs and designing effective interventions [9, 18].

Qualitative research focuses on understanding participants’ perspectives and the contexts in which these perspectives or views are situated [19]. It is therefore a valuable way to identify factors that affect safety behaviour and inform the design of educational interventions. However, the authors could not identify any previous qualitative studies that have examined workers’ perceptions about factors affecting safe work behaviours in the petrochemical industry. Previous research has focused on quantitative assessment and methods to identify risk factors leading to unsafe work behaviour, and consequently work-related accidents [14, 20]. Loosemore and Malouf [21] reported that the main cause of occupational injuries and incidents in industrial settings is a lack of effective safety training interventions. Unfortunately, however, the absence of qualitative evidence on the factors affecting workers’ safety behaviour significantly constrains the development of effective safety training programs in the petrochemical industry. This study addresses this gap by using qualitative content analysis to identify factors that workers believe are associated with unsafe work behaviours in the petrochemical industry.

A small number of qualitative studies has, however, highlighted the importance of psychosocial and working environments, management systems, inadequate and outdated occupational health and safety training, safety communication strategies as major factors contributing to safety promotion in the petrochemical industries [22–24]. A mixed-method, qualitative study was performed to explore occupational health and safety practices in a Malaysian petrochemical company. This study highlighted safety culture and the impact of psychosocial risks on occupational health and safety outcomes. Psychosocial factors and work-related stress are considered major occupational health concerns in the petrochemical industries in Malaysia. A policy review was undertaken and interviews were conducted with government officials, company managers, and key experts to explore their perceptions and views. Inadequate and out of date occupational health and safety (OHS) training was reported by middle management [22]. Another qualitative interview study was conducted in a Norwegian petroleum company to explore the various ways the Health, safety and environment (HSE) concept is used and understood by managers and employees in one company in order for other industries to develop strategies, methods, and actions to promote the HSE performance [23]. Nedzamba’s (2018) findings indicated that effective safety training and the establishment of efficient safety systems are likely to increase in the likelihood that workers will regularly report incidents and near-misses [24].

The findings above suggest it is important to further explore the nature of safety risks in the petrochemical industry and the types of safety training programs that are likely to reduce injuries and dangerous incidents. This study addresses this gap by using qualitative content analysis to identify factors that workers in the industry report to be associated with unsafe work behaviours.

Given the increasing number of work-related incidents in the Iranian petrochemical industry, it is important to more fully understand workers’ perceptions of the factors affecting safe behaviour and how to promote and maintain it. The attitudes and experiences of workers, supervisors, and safety officials, in particular, are an important source of evidence for identifying the factors associated with the occurrence of dangerous incidents [25]. Accordingly, the aims of this study are to (a) explore workers’, supervisors’ and safety managers’ perceptions of safety in a petrochemical company in Iran, and (b) identify factors that discourage safe behaviours.

## Methods

### Participant recruitment and eligibility criteria

To obtain a broad cross-section of worker opinions and experiences, multi-stage sampling was used [26]. This approach involves a combination of two or more sampling techniques. By combining sampling methods at different stages of research, researchers can increase confidence that they are mitigating biases and engaging hard-to-reach, vulnerable participants [27]. In this study, purposive sampling was supplemented by snowball sampling to enhance recruitment. Purposive and snowball sampling methods were selected because the research team considered the combination of the two was the most practical means to secure a representative sample of company employees [26].

Members of the company’s Safety, Health and Environment unit, who were not part of the research team, assisted with the sampling process. They invited workers, supervisors, and safety managers from various occupational groups working in the operations department and the maintenance and repair department who had experienced accidents and injuries or had witnessed colleagues’ accidents to participate in the study (purposive sampling). Workers were eligible to participate if they had worked in the petrochemical industry for at least 2 years. All workers in the petrochemical industry were male. During the interviews, respondents identified employees who had information about workplace accidents in the company and were key informants (snowball sampling). These employees were also invited to participate in the study. Before the start of each interview a member of the safety staff introduced the participant to the first author, who provided clear verbal information about the study [26].

Based on the company organization chart, 1180 permanent employees were employed in this petrochemical company. Twenty male participants were recruited in the study. They were aged 27 to 47 years (M = 36.38, SD =5.24) and had education beyond undergraduate diploma (*n* = 16, 80%). The participants had work experience 3 to 26 years (M = 13.11, SD =5.99). The majority of participants who had experienced a work-related accident had more than 10 years of work experience. All participants were classified into three categories including workers (*n* = 15, 75% of total), supervisors (*n* = 3, 15% of total) and safety staff members (*n* = 2, 10% of total).

### Semi-structured interviews

Interviewing offers a means to ask participants why they made the decisions they did, thereby providing useful insights and information about intent and actual behaviour [28]. Semi-structured interviews were conducted to gain detailed understanding of factors associated with unsafe behaviours in the company [26]. The interviews took between 30 and 45 min. Probe questions were used when answers were vague or ambiguous or to obtain more specific or in-depth information. Conducting the interviews and analysing qualitative data occurred through an iterative process, such that data from earlier interviews were allowed to influence the content of later interviews [29]. All respondents were asked identical questions in the same sequence, but the interviewer probed inductively on key responses [26]. Sometimes the respondents provided information on new areas and these were included in subsequent interviews. This process enables the researcher to gain insight deeper into the data, while continuing data collection [30]. Data were collected until no more new themes emerged from the data and the interviewer became confident that data saturation had been achieved [31–33]. Data saturation is reached when the final interviews do not reveal any new themes or introduce new elements of an existing theme. A total of 20 interviews were conducted before saturation was reached. The 20 participants included workers, supervisors and safety staff members.

The sample size and amount of data generated from the interviews was ‘satisfactory’ within the range suggested as being sufficient for saturation to be reached [31].

### Data collection

The interviews were conducted between May and July 2017 at mutually convenient time and private areas at the participants’ workplaces. The interview questions were classified into three categories:

1. How safe do you feel in your workplace?
2. Have you experienced workplace accidents directly yourself or have you witnessed accidents involving your colleagues in the workplace?
3. What are the main factors contributing to the occurrence of those accidents?

The interviews were audiotaped and a summary of the key issues discussed in each interview was then sent to each participant to ensure that the researcher had accurately interpreted that participant’s comments (a ‘member check’) [34]. Interviews were transcribed verbatim in Persian language. The transcripts were also translated from Persian into English for qualitative data analysis [26]. Data collection was undertaken by the first author, an Iranian, who also transcribed and translated all the interview data to ensure consistency. Any identifying information in the transcripts was removed prior to data coding.

### Data analysis

This is an exploratory study in which the data and interpretation are grounded in the views and experiences of the participants. Consequently, the researchers deliberately did not impose preconceived, theory-based notions about which codes, categories or themes would emerge. Instead, the data were allowed to drive these interpretations [35, 36]. Conventional content analysis was not the only qualitative approach that could be used to achieve the goals of the current research, but it was deemed the most appropriate to describe a little studied topic while staying close to participants’ words and perspectives, in an effort to elucidate potential interventions for promoting safe behaviour.

Conventional content analysis was used to interpret the content of text data through a systematic classification process involving coding and identifying themes [35]. A team of six coders (four in Iran, two in Australia) reviewed the transcripts and conducted analysis in both languages. Open coding was carried out to allow codes to emerge from the qualitative data and avoid codes based on predispositions of the authors. Codes were repeatedly discussed and revised by the authors to achieve consensus and memos written to explain the analysis [37]. To increase inter-rater coding reliability, only the codes and themes that were validated by at least two of the three coders (the first author, an Iranian and two Australian authors) were included in the results. Immersion in the data is an important first stage in the analysis process during which transcripts are read and re-read many times to become completely familiar with the data. Repeated reading and re-reading of transcripts without coding helps identify emergent themes from the data without losing the connections between key concepts and their context. Content analysis was performed using MAXQDA (Ver. 2018) software to facilitate and document the coding process and retrieve codes afterwards. It should be noted that while software can assist researchers to organise qualitative data, computer software for qualitative analysis does not analyse data and the researcher makes decisions about coding participants’ responses, and the relationships between codes, coding categories and broader themes. MAXQDA allows the researcher to upload raw data, such as transcribed interviews, that can be then coded and cross-referenced in ways that facilitate organising the data for easy retrieval [26].

This study employed the approach to qualitative content analysis described by Graneheim and Lundman [38]. This approach consists of the following elements: units of analysis, meaning units, condensation of meaning units, and development of codes, categories and themes. One of the most basic decisions when using content analysis is selecting the unit of analysis. Whole interviews or observational protocols are considered to be units of analysis. In the second step, the interview text is divided into smaller units called meaning units. A meaning unit - which could be words, sentences or paragraphs - contains aspects, words or statements that relate to the same central meaning. In the third analysis step, condensation, meaning units are shortened while still preserving their core meaning. In the fourth step, codes are developed as descriptive labels for the meaning units. They are tools to help researchers reflect on the data in new and different ways. The fifth step is to sort codes into categories that answer the question, “What?”. In other words, a category is formed by grouping together those codes that are associated with each other through their content or context and belong together. A theme can be seen as expressing the underlying meanings together in two or more categories. The final step of data analysis is the creation of themes. A theme answers the question, “How?”. Therefore, theme names include verbs, adverbs and adjectives and are very descriptive [26].

### Ethics

The Medical Research Ethics Committee of Tarbiat Modares University in Iran approved the study protocol (Approval ID: IR.TMU.REC.1395.503). All participants provided written consent to participate in the study, were advised that data were going to be anonymised, securely stored, and analysed for publications. They were advised that participation was voluntary, and they were free to leave the study at any time.

## Results

Table 1 demonstrates the classification of emerging codes, categories and themes from the semi-structured interviews during the content analysis. Results are presented in detail within this framework below.

**Table 1:** Classification of themes, categories and codes according to the content analysis

Theme	Category	Code
Poor direct safety management and supervision	Ineffective safety system	- Inadequate safety training for workers and safety staff - Inappropriate quality and design of personal protective equipment - Managers not carrying their safety management role effectively - Sub-standard or inappropriate safety equipment promotes accidents - Supervisors not emphasizing and prioritizing safety - No separate allocation of funds to improve safety
	Poor safety monitoring	- Managers’ lack confidence to deal with safety hazards or issues - Safety officers not enforcing safety practices and lacking experience and authority - Inadequate number of safety officers on site - Irregular safety inspections - Contractors not prioritizing safety equipment and training
Unsafe workplace conditions	Unsafe physical environment	- Excessive noise impairing concentration - Use of worn-out and defective equipment - Working in high-temperatures
	Unsafe psychological environment	- Work-related fatigue - Excessive workloads - Delayed salary and wage payments reducing safety incentives - Poor social working environment - Inadequate pay and financial detract from focus on safe behaviour - Low safety motivation - Little encouragement for workers to contribute to safety - Work-related stress - Separation from family - Low level of organizational commitment
Workers’ perceptions, skills and training	Workers not skilled enough to deal with safety issues	- Lack of experience and skills in dealing with hazards. - Taking greater risks when doing common tasks - Need for more sharing of previous experiences with hazards - Hazards becoming ‘normalized’ over time - Inadequate safety orientation for new workers - Use of untested work practices
	Active errors	- Workers distracted by making errors - Not seeking help when minor incidents occur - Workers ignoring safety instructions for machinery - Low level of safety efficacy - Unrecognised health conditions contributing to errors
Broader organisational factors	Unsafe management culture	- Prioritizing work outcomes over safety - Management purchases low-quality safety products and equipment - Condescending safety supervision and bullying
	Organisational impact on workers’ safety	- Lack of attention to workers’ emotional and mental needs - Lack of organizational safety training at appropriate levels - Workers underestimating routine hazards - Poor organisational safety culture influencing workers’ behaviour - Inadequate staffing - Incidents may occur even when workers behave safely

NOTE. This table gives an overview of the themes and categories identified in the interview data. Every category is described with extracted codes from the interviews

MaxQDA provides tools for analysing and synthesising qualitative data. Table 2 describes the codes included in the *“Ineffective safety system”* category of the *“Poor direct safety management and supervision”* theme (theme 1).

**Table 2:** Frequencies of the codes of “Ineffective safety system” category of the “Poor direct safety management and supervision” theme (theme 1)

Codes of the “Ineffective safety system” category	Frequency	Percentage	Percentage (valid)
Inadequate safety training for workers and safety staff	10	52.63	55.55
Inappropriate quality and design of personal protective equipment	7	36.84	38.88
Managers not carrying their safety management role effectively	5	26.31	27.77
Sub-standard or inappropriate safety equipment promotes accidents	5	26.31	27.77
Supervisors not emphasizing and prioritizing safety	4	21.05	22.22
No separate allocation of funds to improve safety	4	21.05	22.22
Interviews with code(s)	19	94.12	100.00
Interviews without code(s)	1	5.88	–
Analysed Interviews	20	100.00	–

### Theme one: poor direct safety management and supervision

Poor direct safety management and supervision was mentioned as a factor influencing unsafe behaviours. It comprised two categories: ineffective safety system, and poor safety monitoring.

#### Ineffective safety system

Ineffective safety system was cited as the extent to which supervisors and managers put safety as main priority regardless of administrative pressure (e.g., supervisors not emphasizing and prioritizing safety)” (P11 refers to participant 11):

*P11: “I think that supervisor’s positive attitude toward the safety leads to a better safety compliance on site. When I as a supervisor ignore safety regulations or disregard reporting the hazards, in essence, safe work practices have been given lesser priority in our workplace.”*

Participants mentioned that employers must provide adequate and appropriate protective personal equipment to workers exposed to risks. Management often overlooks personal protective equipment (PPE) as a key to worker safety, for example:

*P3: “Our duties are such that we need to be very careful, when we use personal protective equipment. We are very cramped for space and the precision is reduced and the incidence of accidents may even increase. I do not wear my safety helmet at all times because due to the poor design of helmets, it reduces visibility and precision while working, especially when it comes to the hazards that may arise from the items above head height.”*

Inadequate safety training for workers was the most frequently reported cause of work-related accidents. Workers reported they did not have the knowledge, confidence or skills to recognize potential hazards:

*P14:* “*… [lack] the specific skills and knowledge required for workers to perform their specific tasks correctly. Workers here just attend general safety courses at the beginning of their employment and there are no specialized training safety courses according to our job health and safety requirements. We cannot keep displaying our confidence in dealing with health and safety issues and addressing these challenges. You know what I’m saying? Well-trained workers have the capacity to predict potential hazards, work safely and even teach the newer workers.”*

*P6: “I mean untrained and inexperienced workers are not able to meet their job performance standards, they are more likely to experience work-related stress and are susceptible to workplace accidents. Managers and supervisors who ignore their responsibilities to provide adequate and appropriate job-related health and safety training for workers could face an increase workplace accidents and injuries.”*

#### Poor safety monitoring

Poor safety monitoring was identified as contributor to inhibiting safe behaviours. Some participants referenced a lack of the authority and experience of the safety unit safety (e.g., Safety officers not enforcing safety practices and lacking experience and authority), for example:

*P1: If we do not use personal protective equipment or overlook requirements for safety, safety officers will not blame us because our work experience is more than them.*

Many participants expressed that periodic inspections were not carried out to identify hazards that may cause safety issues at work (Irregular safety inspections):

*P18: Specialists do not audit our workplace regularly to identify potential hazards and assess the risks. If inspectors attended the site periodically (at least once every 3 months), they could assess potential risks that may result in workplace accidents.*

### Theme two: unsafe workplace conditions

Two categories of unsafe workplace conditions that were mentioned as significant determining factors in creating risks for workers were unsafe physical environment and unsafe psychological environment.

#### Unsafe physical environment

Unsafe physical environment mainly referred to ventilation, temperature, noise, heat, humidity and other changeable environmental factors affect industry safety (e.g., working in high temperatures), for example:

*P16: I give all protective clothing and equipment such as safety shoes, face shields, gloves and so on to my workers and I want them to use this equipment, but in a high temperature and pressure area they cannot work with safety equipment for more than five minutes (Now that I’m talking to you, the temperature of the site is 40 °C). This is what I’ve experienced so far, and I cannot force them more than that.*

According to participants, if a tool or equipment was defective it would not be taken out of service for repair (use of worn-out and defective equipment):

*P10: A barrier to safe behaviours would be that equipment and tools are very worn-out and defective. Due to the defective and old machinery and equipment on the site, we cannot practically carry out many of our duties in compliance with the safety principles.*

#### Unsafe psychological environment

Beyond unsafe physical environment, unsafe psychological environment was also referred to as a hidden danger that petrochemical industries are facing. Lack of motivation, work-related fatigue, low appreciation or gratitude towards co-workers, work-related stress due to heavy workload, no sense of belonging to the organisation and preoccupation with inadequate pay (inadequate pay and financial detract from focus on safe behaviour) are perceived to detrimentally affect workers’ safe behaviours at work:

*P12: I think it would be important for all workers to be focused on their duties while at work and have no financial concerns … unfortunately, when I’m working, my thoughts are involved in spending on living costs and I cannot focus on my work.*

In addition, participants mentioned appropriate treatment by the organisation such as involving workers in decision making, talk on safety visions is related to promote safety motivation and will encourage workers’ safe behaviours. As alluded to above, little encouragement for workers to contribute to safety may also contribute to unsafe behavioural patterns:

*P14: Our managers do not pay much attention to the workers’ viewpoints which undermine the workers’ self-confidence. When we propose our perspectives on safety decisions, management does not accept our suggestions.*

### Theme three: workers’ perceptions, skills and training

Workers’ perceptions, skills and training were mentioned also may be a contingency factor affecting workers’ safe behaviours mainly include two categories: workers not skilled enough to deal with safety issues and active errors.

#### Workers not skilled enough to deal with safety issues

According to participants’ views, in order to improve safe behaviours, workers need to display their readiness and confidence when dealing with safety challenges, and share their experiences to prevent similar future events, focusing on their adaptation to change their behaviours in accordance with environment requirements (inadequate safety orientation for new workers):

*P14: It seems that due to different working and environment conditions in southern Iran’s petrochemical industries, new workers’ adaptation to the work environment is a time-consuming process. These workers are more likely to be injured than experienced workers and need proper orientation to be safe in the workplace.*

As a result, taking greater risks when doing common tasks and risk behaviour based on experience represents a major barrier to effective safe behaviour at work. For example:

*P10: It’s actually a general problem that when I have worked at height, my confidence to not wear a safety belt is often problematic, because it’s my daily task. And that happens quite often. Then I just don’t think need to wear it.*

*P17: “A lot of times in our work units … doing routine job tasks quickly while talking to colleagues or thinking about the problems in life without considering safety regulations and procedure can lead to a mistake, quite often slow down the response to preventing incidents … because we assume that we are quite expert in our daily tasks.”*

#### Active errors

According to interviews conducted with supervisors, those human errors that had immediate consequences were usually caused by operational personnel such as the workers of the operations and repair department. These errors were the direct cause of the incident in the events leading to the accident. Some workers expressed that they were distracted by making errors at work due a variety of concerns such as financial problems, work-family issues and so on which can be a major cause of occupational injuries:

*P3: My worries about something made me forget to close the passage that day … I lost my concentration and made mistakes because I thought about that all that day.*

Many participants agreed that minor incidents helped to deal with serious incidents. Additional problematic situation was the low level of safety efficacy. Workers felt that when they met safety challenges, their ability to engage in safe behaviours at work is limited:

*P17: I think that many of the company’s workers wouldn’t be able to keep their confidence and belief to face unpredictable challenges and situations. Therefore, they cannot enact safe behaviours in the face of hazards.*

A number of participants reported that unrecognized health conditions contributed to errors and hazards. Chronic diseases such as diabetes, hypertension, heart disease and osteoporosis were the leading cause of occupational accidents, for example:

*P12: … as you probably know, workers who are working in the company have been suffering from osteoporosis and their bones have been weakened by osteoporosis... But caring for their health is not one of company’s safety policies, which increases workers’ exposure to workplace accidents.*

### Theme four: broader organizational factors

In regard to situational factors, the majority of the participants mentioned that broader organisational factors could lead to the occurrence of unsafe behaviours. In addition, unsafe management culture, and organisational impact on workers’ safety were identified as main categories of broader organisational factors.

#### Unsafe management culture

In relation to the unsafe management culture, the most commonly cited problems related to negative management approach to provide high quality product for workers, and the low priority that management puts on safety, especially when safety goals conflicts with the production (prioritizing work outcomes over safety):

*P5: Well If the condition presents a risk of danger and serious injury or a device stopped working properly, our employer has asked us to work without he eliminates the hazards. And in this moment, there is no right for us to refuse to work in these unsafe situations.*

Participants also reported management attitude towards pinning blame on certain individuals rather than solving safety problems when a safety incident occurs (condescending safety supervision and bullying):

*P4: If we make mistakes, our supervisor blames us in public. When a safety problem occurred, our supervisor made threats to workers and workers cannot criticize him for his behavior.*

#### Organisational impact on workers’ safety

Organisational impact on workers’ safety was achieved through influencing workers’ behaviour by organisational safety culture. Participants noted that workers infrequently underestimate the risk of duties that they perform regularly that then could lead to workplace accidents and injuries. (workers underestimating routine hazards):

*P18: When workers are exposed to relatively constant and well-known risks in their work activities for a long time, they will underestimate the risk of occupational hazards and this will provide the basis for the incident.*

Many interviewees expressed that if a supervisor considers workers’ needs and empathizes with their problems, pays attention to their welfare, provides appropriate training safety training for workers; and then it is likely that workers will be encouraged to work safely (e.g., lack of attention to workers’ emotional and mental needs), for example:

*P11: Well, basically our supervisors in this organisation, not as a mentor, but as a head and superiors, deal with workers and do not care about our psychological needs and desires. If they pay attention to our problems and we earn respect from the organization, we will also be mutually committed to organisation’s safety.*

With regard to aspects related to inadequate staffing, safety professionals emphasized that in petrochemical industries workers’ awareness and ability to recognize potential safety hazards is required and safety should be strictly monitored and managed at all levels to minimize and eliminate risks:

*P13: There are not enough young workers in the organisation. So, we do not have enough people to cover response to emergencies in the rotating shifts. Particularly when a colleague is absent and others are not trained in the skills demanded of emergency response plans.*

## Discussion

The purpose of this study was to explore workers’, supervisors’ and safety managers’ perceptions of safety at work in a petrochemical company in Iran and identify the factors that discourage safe behaviours. Using a qualitative approach, and conventional content analysis to interpret the data, this study found the most commonly cited the contributory factors to exhibit unsafe behaviours were: 1) poor direct safety management and supervision, 2) unsafe workplace conditions, 3) workers’ perceptions, skills and training, and 4) broader organisational factors.

The findings are valuable for researchers, safety specialists, and enterprises, as they often overlook potential hazards at workplace. Some results are consistent with the findings of previous studies [9, 39–41], including the challenges of using PPE, the need for adequate and up-to-date safety training, high workloads contributing to safety procedures not being implemented and managed in an effective manner that facilitated their use in industrial settings. However, there has been limited safety research in the petrochemical industry aimed at identifying factors discouraging safe work behaviour, such as the studies by Cheng and colleagues [9], Xue and colleagues [42], Min and colleagues [43], Hong and colleagues [44].

Cheng and colleagues [9] reported that the vast majority of accidents in petrochemical companies are associated with inappropriate and inadequate safety training. Appropriate and adequate training programs have a direct effect on workers’ safe behaviour. Therefore, to control potential hazards and reduce or prevent accidents, regular educational training and effective safety interventions should be provided to reinforce workers’ knowledge and raise their awareness about potential hazards in the workplace [14, 45]. In addition, managers and employers should hold regular site inspections and meetings to check for high-risk work hazards. These strategies promote the safety conduct of workers and help prevent incidents from recurring [9, 41].

Several studies have focused on the use of PPE [45–48]. Both individual and organizational factors can affect whether workers use PPE or not. Probably more importantly, use of PPE is discouraged if it is ill-fitting, ineffective or impedes vision and situational awareness. Similarly, if production pressures are high and PPE slows work, it is likely to be discarded. Underestimating work hazards, over-confidence with routine tasks and increased workload may also discourage workers from PPE compliance. Organisational factors, such as prioritising safety and the importance and value the organization places on safety within the workplace influence the use of PPE [9, 46]. It is also imperative that employers provide high-quality PPE that meets recognised standards [49]. Some participants noted a lack of availability of PPE was a reason for not using it.

The findings also offered important new insights into safety in the petrochemical industry. For example, in relation to poor direct safety management and supervision, the participants referred that managers did not keep displaying their confidence and skills when dealing with safety issues. The lack of authority and power of the managers, no separate allocation of funds to improve safety and not providing adequate safety training at appropriate levels were also perceived as factors shape the context that contributes to unsafe behaviours and error occurrence. These findings are consistent with previous evidence that management should provide funding sources and adequate support to achieve safety goals [50]. The ability of supervisors/managers to tackle safety issues when they arise and to display confidence and expertise when meeting safety challenges is often seen as a critical factor for promoting safety in high risk settings. Generally, managers need to have skills and traits in relation to safety. Managers’ concern for safety can function as a frame of reference for the workforce to guide appropriate task behaviours and can reflect managers’ commitment to workplace safety [51].

Another significant finding relates to categories of the unsafe workplace conditions. Participants reported that their concentration was impaired by excessive noise levels and working under high temperature was also perceived as detrimental to working safely. Indeed, features of poor social working environments may operate as stressors and have been linked to perceptions of safety. Our findings provide additional support to existing findings that emphases the importance of safe workplace environment. For example, Zhang and colleagues [52] identified poor working conditions as ranking second in terms of factors in their study that influence work safety.; only in a good work environment will the influence of other contributory factors of unsafe behaviours be reduced to a minimum. The importance of interactions between managers and workers was highlighted by this research. According to Peterson and colleagues [53], conscientious senior managers are more likely to focus on the social relationships that managers or supervisors establish with their subordinates and create the positive working environment between them.

Work environment factors, such as resourcing levels, communication among staff, and working system, have the greatest effect on workers’ sense of belonging to the organisation and contribute to positive safety behaviours [54]. When an organisation has a positive work environment, the levels of worker engagement in safety activities could possibly be enhanced [55]. The staff can cope better with unexpected situations and handle challenges more effectively when they meet safety challenge in their job tasks. It motivates employees to continue their safety work and maintain their commitment [56].

With regard to workers’ perceptions, skills and training, the majority of participants reported that doing common tasks and duties could contribute to higher risk taking. Empirical studies provide evidence that workers rely on their experiences to form risk perceptions [48, 57, 58]. Workers may perceive they are not at risk while doing routine tasks. Workers often perceive risks in connection with new task demands and non-routine rather than with their routine tasks. As a result, risks associated with common responsibilities are frequently underestimated [59, 60]. These qualitative findings are also in line with results of a recent study that indicates identification of hazards is the primary phase of the risk assessment process. Hazard identification, assessment, and control is a process to minimize the possible work-related injuries. When potential hazards have been identified in workers’ duties, the risks associated with those hazards must be examined [61]. Based on the evidence, providing complete information about risks associated with working tasks is one of the most effective strategies for regulating workers’ safe behaviours, because workers often overlook risks when performing their work duties [62]. Also, the finding that inadequate safety orientation for new workers may contribute to working in an unsafe manner is consistent with previous studies [63–66]. New workers in the petrochemical industry are unfamiliar with the required precautions, working conditions and safety hazards, but may be fearful about asking questions. The present findings highlight the importance of considering supervisors as a potentially important source of improvement in new workers’ safety outcomes. This explanation implies that new workers’ risk-taking orientation due to different working conditions, which includes behaviours such as taking risks to get work done faster, was positively associated with workplace accidents [63]. New workers may not be aware of the hazards in their new workplace or locations that have different hazards and they may feel pressured to perform tasks quickly to keep up with experienced workers or to adapt to their new work environment with little guidance. Tucker and colleagues [64] reported that employers should provide young and new workers with adequate training as part of initial and ongoing job training.

The finding from this study regarding the need for greater sharing of previous experiences with hazards is as a key element that has a greater impact on safe behaviours of the workers which is in keeping with findings from a Korean study [54] which reported that it is important for employees to share safety issues and personal experiences in their daily work and make suggestions on how to improve safety in the workplace.

In relation to broader organisational factors, some participants remarked that workers exposed to condescending safety supervision and workplace bullying are more likely to engage in risky and unsafe behaviours. These findings have been included in a British study [67] in which safety professionals reported their experiences of workplace bullying and the extent to which they had been pressured to make risk based decisions. Employees who had experienced workplace bullying were more likely to engage in a broad range of dangerous and unsafe behaviours than those who had not [67]. Several participants in the present study noted that their supervisor uses a condescending tone and makes threats to workers when safety problems occur and workers cannot criticize his behaviour, which causes a conflict between the workers and the supervisor. Many previous studies have linked bullying from supervisors with negative organisational consequences such as engaging organisation and employees in unsafe practices and increasing on workplace errors among employees in industry contexts. Work-related bullying plays a major part in occupational health impairments and safety issues that could result in long-lasting damage [68]. Workplace bullying may cause health impairment outcomes, such as quantitative and qualitative job insecurity and loss of self-esteem and self-confidence that leads to unethical practices and occupational injuries [69]. In concordance with other studies [70], nurses who reported a higher frequency of perceived workplace bullying were found to have higher turnover intention, perceived more adverse outcomes to patient safety —for example, medication errors.

Some of the participants described how inadequate staffing could place workers at high risk for occupational accidents because it does not cover response to emergencies in the rotating shifts. This finding is in line with the responses from participants in a secondary analysis of data from the 2016 AWHONN nurse staffing survey, consequences of inadequate staffing can be quite serious and put patients at risk for preventable harm due to missed care [71]. The results of a Korean cross-sectional survey suggested that nurses were overloaded at least in part by a high patient-to-nurse ratio. This overload was demonstrated by working overtime beyond their contracted hours and by leaving care activities undone. Inadequate nurse staffing and a heavy workload were associated with poor or fair patient safety and lower quality of care [72].

## Limitations and recommendations

Due to the exploratory nature of the qualitative methodology, the findings in this study should be interpreted with care, recognising several limitations. The analysis is based on twenty interviews. This approach highlighted workplace safety risks that would not have been identified by approaches relying on a priori identification of the variables to be studied, and also provided detailed subjective reports illustrating how those factors are perceived to affect workplace safety. However, the number of interviews conducted does not allow for straightforward generalization beyond the sampled organisation or industry. The detailed qualitative analyses were conducted on transcripts from 20 in-depth interviews within one Iranian petrochemical company. The findings may not generalise to other industrial settings, which are likely to have different organizational and physical risks. As a result, these qualitative findings may not be transferable to new situations or populations, and replication with samples drawn from other contexts is important. Furthermore, as all workers in the petrochemical company were men, the findings only represent male participants’ perspectives. While the study sheds light on the factors that discourage safe behaviours, future studies research would benefit from identifying petrochemical facilities that employ women and endeavouring to sample female workers. Future studies could systematically sample comparable male and female participants to compare their experiences. While this study included extensive data from interviews, it should be noted that participants were recruited from a petrochemical company that has had the highest incidence rates of occupational accidents due to unsafe behaviours. It is possible that the experiences reported by the participants did not reflect the experiences of workers employed in other petrochemical companies in Iran.

## Conclusions

The present study indicates that various types of personal, behavioural and environmental factors may discourage petrochemical workers’ from behaving safely. The responsibility of the individual is important to reduce or eliminate these risk factors for unsafe behaviours, but the role of management is vital to provide resources for safety work best promote workers’ commitment to safety. The resources include time for safety work, PPE and safety procedures, appropriate training and support provided by superiors, co-workers and safety professionals. Quantitative research studies are required to confirm our observations and expand the evidence to industrial outcomes. Work is in progress to examine complex relationships among the identified constructs. In future reports, we will present the findings as well as theoretical models that have been used to explain and predict safe behaviour in the workplace, both in the petrochemical industry and more generally to identify a theoretical model that fits with qualitative data and provides a suitable organising structure for using in petrochemical industries.

This study provides a deeper understanding of workers’, supervisors’ and safety managers’ perceptions and views and recommendations for addressing factors affecting safe work behaviours in the petrochemical industry that could be used to inform the design of educational interventions. Previous studies have been carried out to understand the key factors affecting safety in industrial settings with quantitative methods while workers’ perceptions are often overlooked. These insights offer important context to overcome the barriers that workers face to performing their duties safely.

## Data Availability

The qualitative datasets generated and analysed during this study are not publicly available and cannot be shared due to participant confidentiality, as participants are potentially identifiable from the information contained in the data.

## Abbreviations

OHS: Occupational Health and Safety
HSE: Health, Safety and Environment
PPE: Personal Protective Equipment
